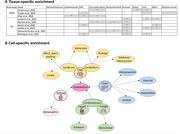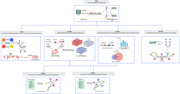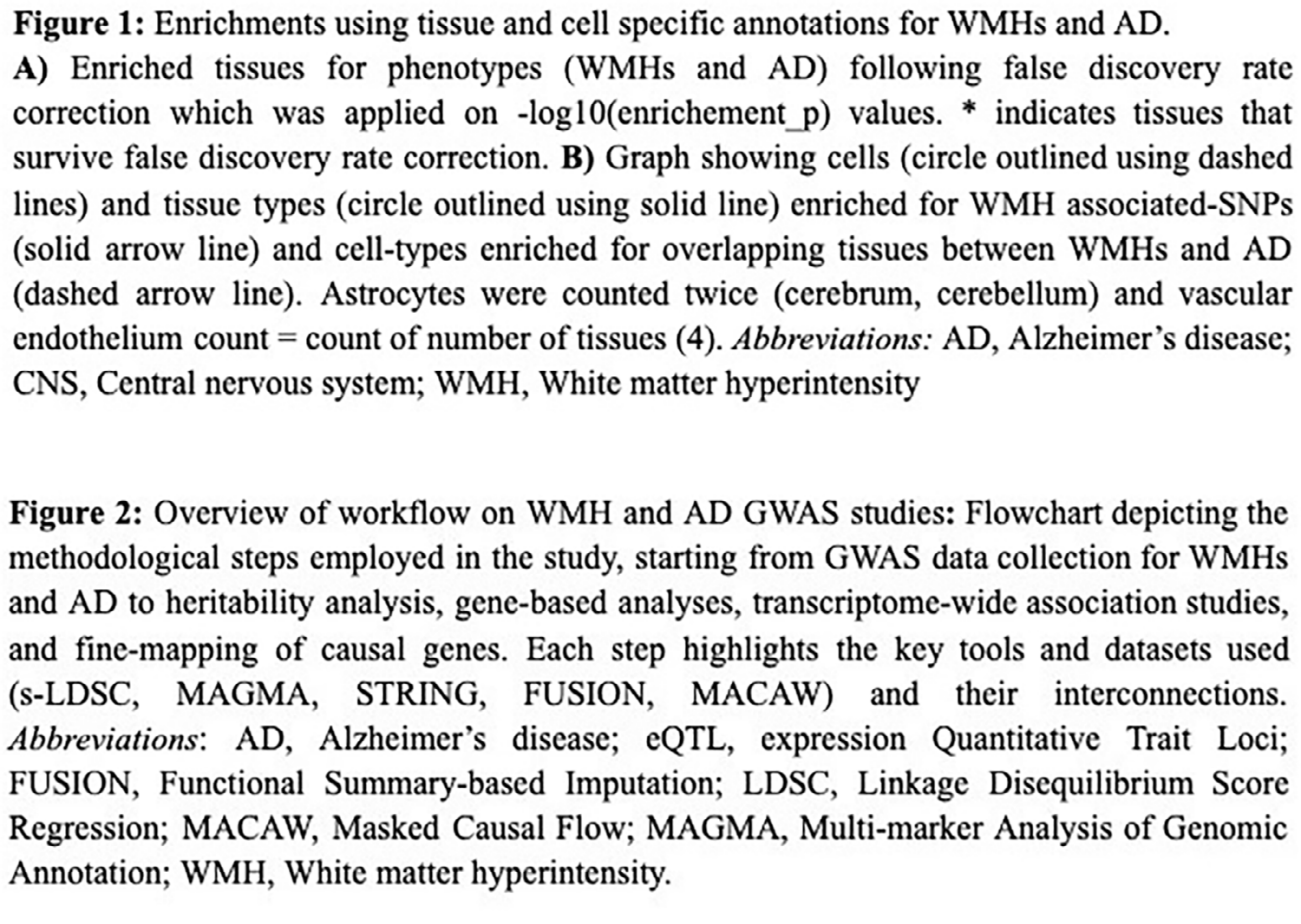# Genetic and Causal Insights Into White Matter Hyperintensities Across the Brain‐Body Axis

**DOI:** 10.1002/alz70862_110035

**Published:** 2025-12-23

**Authors:** Manpreet Singh, Kimia Shafighi, Flavie E. Detcheverry, Gabrielle Dagasso, Fanta Dabo, Ikrame Housni, Sridar Narayanan, Nils D. Forkert, Sarah A Gagliano Taliun, Danilo Bzdok, AmanPreet Badhwar

**Affiliations:** ^1^ University of Montreal, Montreal, QC Canada; ^2^ Centre de recherche de l'Institut universitaire de geriatrie de Montreal (CRIUGM), Montreal, QC Canada; ^3^ Institute of Biomedical Engineering, University of Montreal, Montreal, QC Canada; ^4^ Multiomics Investigation of Neurodegenerative Diseases (MIND) Laboratory, Montreal, QC Canada; ^5^ McGill University, Montreal, QC Canada; ^6^ University of Calgary, Calgary, AB Canada; ^7^ Université de Montréal, Montréal, QC Canada; ^8^ Centre de recherche de l'Institut universitaire de gériatrie de Montréal (CRIUGM), Montréal, QC Canada; ^9^ Multiomics Investigation of Neurodegenerative Diseases (MIND) Laboratory, Montréal, QC Canada; ^10^ Montreal Neurological Institute‐Hospital, McGill University, Montreal, QC Canada; ^11^ Neurology and Neurosurgery, McGill University, Montreal, QC Canada; ^12^ McConnell Brain Imaging Centre, Montreal Neurological Institute, McGill University, Montreal, QC Canada; ^13^ Hotchkiss Brain Institute, University of Calgary, Calgary, AB Canada; ^14^ Montreal Heart Institute, Montreal, QC Canada; ^15^ Université de Montréal, Montreal, QC Canada; ^16^ Mila ‐ Quebec Aritificial Intelligence Institute, Montreal, QC Canada

## Abstract

**Background:**

White matter hyperintensities (WMHs), visible as bright regions on T2‐weighted FLAIR MRI, are frequent with age and elevated in Alzheimer’s disease (AD). Representing axonal damage, demyelination, and edema, WMHs are driven by vascular mechanisms, including endothelial dysfunction and impaired cerebrovascular autoregulation. WMHs also exhibit strong heritability (55–73%), with overlapping genetic pathways shared with AD. Emerging evidence suggests systemic factors across the brain‐body axis influence WMHs, yet these contributions and their genetic overlap with AD remain underexplored. Our study investigated genetic underpinnings specific to WMHs and those shared with AD by assessing partitioned heritability of WMHs and AD across the brain‐body axis with SNP level tissue‐ and cell‐specific annotations; identifying genes associated with WMHs and AD through integration of gene expression data, establishing causal links between SNP‐level findings and imaging‐derived phenotypes (IDPs), particularly structural variations in regional brain volumes.

**Method:**

Partitioned heritability was assessed using stratified‐linkage disequilibrium score regression (sLDSC) on GWAS summary statistics (*N* = 3 WMH studies; *N* = 6 AD studies) using human A1) tissue level annotations (*N* = 10) and A2) continuous cell‐specific annotations (*N* = 64). MAGMA and FUSION analyses highlighted genes associated with WMH and AD for further bioinformatics analysis (using human protein atlas (HPA) and STRING database). MACAW (Vigneshwaran et al, 2024) modeled causal relationships between WMH‐associated SNPs (from FUMA analysis) and IDPs (*N* = 172), leveraging directed acyclic graphs to evaluate genetic effects while controlling for confounders (Figure 2).

**Result:**

Tissue‐specific analysis revealed significant enrichment of WMH‐associated SNPs in the CNS, liver, cardiovascular system, and kidneys, while AD‐associated SNPs were enriched in the CNS, connective bone, liver, and immune tissues. (Figure 1). Cell‐specific analysis identified vascular endothelial cells as enriched across WMH‐enriched tissues. MAGMA analysis, combined with HPA analysis, corroborated sLDSC tissue‐level findings. MAGMA and FUSION analyses highlighted genes associated with WMHs (*N* = 39 and 69) and AD (*N* = 291 and 193). MACAW linked WMH‐associated SNP to 172 IDPs, consistently impacting WM hypointensities and regional brain volumes (e.g., left inferior temporal volume).

**Conclusion:**

Our findings highlight systemic multi‐tissue contributions (CNS, liver, cardiovascular system, and kidneys) to WMHs, driven by vascular endothelial dysfunction and shared AD genetics, with SNPs across the body also affecting brain imaging derived phenotypes.